# Species-Level Reconfiguration and Retrospective Bridging Assessment of a Multiplex PCR Panel for Complicated and Recurrent Urinary Tract Infections

**DOI:** 10.3390/cimb48090930

**Published:** 2026-09-11

**Authors:** Moustafa Kardjadj, Itoe P. Priestly, Roel Chavez, Thomas K. Huard

**Affiliations:** 1 MED-US Consulting, LLC., Austin, TX 78734, USA; 2 Soft Cell Laboratories, Hillsboro, OR 97006, USA; 3 Doc Lab Inc., Hillsboro, OR 97006, USA

**Keywords:** multiplex PCR, complicated urinary tract infection, recurrent urinary tract infection, molecular diagnostics, diagnostic agreement, antimicrobial resistance, species-level reporting, assay bridging, uropathogens

## Abstract

Background: Complicated and recurrent urinary tract infections (cUTI/rUTI) remain diagnostically challenging, particularly after antimicrobial exposure and in polymicrobial or fastidious infections. Multiplex PCR may address some limitations of culture, but panel composition requires periodic refinement as epidemiological, resistance, and clinical evidence evolves. This study describes the transition of the DocLab UTM™ multiplex PCR assay from the prospectively evaluated V2 configuration to the redesigned V5 configuration. Methods: V2 pathogen-level agreement with quantitative urine culture and molecular AMR target–phenotypic AST agreement were characterized using 773 baseline specimens from the prospective multicenter NCT06996301 study. V2-to-V5 panel reconfiguration incorporated US epidemiological and AMR evidence, clinical and antimicrobial-stewardship considerations, and species-level reporting value. V5 was assessed retrospectively using 743 evaluable archived specimens from the same NCT06996301 cohort. Pathogen and AMR agreement were evaluated against the original parent-study quantitative culture and phenotypic AST comparators, and directly shared V2–V5 targets were compared within the common paired cohort using McNemar testing with Holm adjustment for multiple target-specific comparisons. Six newly introduced pathogen targets and one internal process-control target underwent limited contrived-urine feasibility testing. Results: V2 showed high agreement for most established pathogen and AMR targets, although lower negative percent agreement was observed for selected composite pathogen groups. V5 separated several composite targets into species-level outputs and modified selected AMR reporting, principally the *qnr* architecture. Across evaluated V5 AMR targets, positive percent agreement ranged from 94.9% to 100.0% and negative percent agreement from 96.6% to 99.6%. After Holm adjustment, paired V2–V5 comparisons identified no statistically significant differences in molecular classifications among the directly shared pathogen or AMR targets (all adjusted *p* > 0.05). All tested spiked-positive and negative contrived specimens were classified as expected under the predefined feasibility conditions. Conclusions: V5 increased species-level reporting resolution while retaining generally high retrospective pathogen and AMR agreement with the original parent-study comparators. Paired analyses did not identify statistically significant directional changes in shared-target classifications. Newly introduced targets require comprehensive validation, and prospective evaluation of the final V5 configuration is needed before conclusions regarding clinical or stewardship benefit can be made.

## 1. Introduction

Urinary tract infections (UTIs) are among the most common bacterial infections and account for substantial outpatient, emergency, hospital, and antimicrobial use. Complicated and recurrent urinary tract infections (cUTI/rUTI) present particular diagnostic and therapeutic challenges because of host comorbidities, structural or functional urinary abnormalities, recurrent antimicrobial exposure, polymicrobial infection, and increasing antimicrobial resistance [1,2].

Conventional urine culture with phenotypic antimicrobial susceptibility testing (AST) remains the established clinical reference method but may require ≥48 h and is affected by prior antimicrobial exposure, polymicrobial specimens, and recovery of slow-growing or fastidious organisms [1,3]. Multiplex real-time PCR can detect multiple organisms and selected antimicrobial-resistance (AMR) determinants without microbial growth, but PCR and culture measure different biological characteristics. PCR detects nucleic acid irrespective of viability; therefore, PCR-positive/culture-negative findings represent diagnostic discordance and should not automatically be classified as molecular false positives [2,3,4].

The molecular UTI diagnostic landscape now includes multiplex approaches with broad pathogen coverage, species-level identification, and selected AMR-marker detection. As these assays evolve, an important challenge is how to refine an established panel as epidemiological, microbiological, resistance, and clinical evidence changes while preserving continuity with evidence generated using earlier configurations. A recent review of molecular diagnostics for complicated and recurrent UTI evaluated the available evidence base and found that the DocLab V2 platform was supported by prospective clinical studies demonstrating clinical utility [4,5,6,7,8,9,10].

Most evaluations of multiplex UTI PCR assays focus on the performance of a single fixed panel configuration [4,11]. Less attention has been given to iterative panel reconfiguration, including separation of composite targets into species-specific outputs, removal of lower-priority targets, consolidation of overlapping resistance determinants, and addition of organisms considered relevant to the intended-use population. Such changes can increase reporting resolution and reduce ambiguity in organism attribution, but they do not inherently establish improved analytical specificity, clinical utility, or patient outcomes.

The present study examines the transition of the DocLab UTM™ multiplex PCR assay from the prospectively evaluated V2 configuration to the redesigned V5 configuration. V2 provides the prospective clinical evidence base [5,6,7,8,9,10], whereas V5 was evaluated retrospectively using archived specimens from the same parent cohort. The study objectives were to:

1. Characterize pathogen-level agreement with quantitative culture and molecular AMR target–phenotypic AST agreement for the clinically evaluated V2 configuration using the NCT06996301 baseline cohort;
2. Describe the structured microbiological, epidemiological, AMR, stewardship, and reporting considerations used to reconfigure the panel from V2 to V5;
3. Evaluate the redesigned V5 configuration using archived NCT06996301 clinical specimens by assessing pathogen-level agreement with quantitative culture, molecular AMR target–phenotypic AST agreement, and paired V2–V5 comparisons of directly shared targets, while separately characterizing the preliminary analytical feasibility of newly introduced targets using contrived urine specimens.

The underlying premise was that panel refinement could increase species-level reporting resolution and reduce ambiguity associated with composite targets. Whether these architectural changes improve analytical specificity, antimicrobial decision-making, stewardship, or patient outcomes remains to be established through comprehensive validation and prospective clinical evaluation.

## 2. Materials and Methods

### 2.1. DocLab UTM™ Assay and V2 Molecular Testing

The DocLab UTM™ assay is a high-complexity multiplex TaqMan^®^ real-time PCR panel intended for the qualitative detection of bacterial uropathogens and clinically relevant antimicrobial-resistance (AMR) determinants directly from urine specimens collected from symptomatic adults with suspected complicated and/or recurrent urinary tract infection (cUTI/rUTI).

The molecular workflow comprised automated nucleic-acid extraction and purification using the KingFisher Flex or KingFisher Duo Prime platform (Thermo Fisher Scientific, Waltham, MA, USA), followed by multiplex real-time PCR amplification and fluorescence detection using the QuantStudio 7 Flex system (Thermo Fisher Scientific). Amplification-curve analysis and software-assisted interpretation generated qualitative target-level results. The assay reports target detection qualitatively rather than as a quantitative measure of organism burden. Target detection therefore indicates the presence of the corresponding nucleic-acid sequence but does not establish organism viability or causation of urinary infection.

For the prospective V2 evaluation in NCT06996301, baseline urine specimens allocated for molecular testing were processed using the V2 DocLab UTM configuration according to the parent-study assay procedure [5,6,7,8,9,10]. Both pathogen and molecular AMR targets were evaluated using the same extraction, amplification, and qualitative interpretation workflow. Pathogen results were compared with the corresponding quantitative urine-culture comparator, whereas molecular AMR results were evaluated against the mapped parent-study phenotypic AST classification.

For V2, pathogen targets with quantification-cycle (Cq) values of 10–32 were classified as detected, whereas AMR targets with Cq values of 10–31 were classified as detected [10]. Signals outside the applicable reporting range were classified according to the parent assay procedure as non-reportable/negative. These Cq ranges were used for qualitative target classification and were not interpreted as organism-specific measures of microbial burden.

### 2.2. Parent Study Population, Specimen Handling, and Culture Comparator

#### 2.2.1. Parent Study Population and Eligibility

The clinical specimens analyzed in this study originated from the prospective, multicenter NCT06996301 study [5,6,7,8,9,10]. Eligible participants were symptomatic adults aged ≥18 years with investigator-suspected complicated urinary tract infection (cUTI) requiring microbiological evaluation and treatment. Parent-study eligibility included protocol-defined urinary symptoms and pyuria together with treatment failure or clinical characteristics associated with complicated infection, including age ≥ 65 years, male sex, pregnancy, recurrent UTI, relevant comorbidities, or functional or anatomical abnormalities of the urinary tract [5]. The participant- and specimen-level eligibility and evaluability criteria relevant to the present analyses are summarized in Table 1.

#### 2.2.2. Urine Collection, Handling, and Transport

At baseline, at least 8 mL of urine was collected using a clean-catch midstream technique or, when clinically indicated, an aseptic collection procedure such as a newly placed urinary catheter. Specimens were refrigerated immediately after collection and maintained at 2–8 °C during transport to the central reference laboratory. Samples were submitted for reference testing within 24 h of collection. According to the parent-study procedures, up to 3 mL was allocated for molecular testing and at least 5 mL for quantitative urine culture.

#### 2.2.3. Quantitative Urine Culture and Pathogen Comparator Classification

Reference urine culture was performed at the central laboratory using quantitative culture procedures defined in the parent study. Specimens were inoculated using a calibrated 1-µL loop onto appropriate culture media, including blood agar and MacConkey agar, and cultures were examined for growth for up to 48 h. For the target-level agreement analyses, a culture-positive result was defined according to the parent-study quantitative criterion of ≥10^5^ CFU/mL for the corresponding reportable organism.

Polymicrobial specimens were not automatically excluded. Where multiple reportable uropathogens were identified, each organism could contribute independently to its corresponding target-level PCR–culture comparison. For voided urine specimens, growth patterns considered potentially consistent with contamination were interpreted according to the central-laboratory procedure using organism quantity, urinalysis findings, and available clinical information. The original culture classification was retained without retrospective modification based on the PCR result.

A PCR-positive/culture-negative observation was defined as detection of a specific pathogen target by PCR when the corresponding organism did not meet the parent-study definition for a positive quantitative culture comparator. No additional molecular assay or independent microbiological adjudication was performed specifically to reclassify these discordant observations. These observations were retained as molecular-positive/reference-negative results in the agreement analyses.

### 2.3. AMR Phenotypic Comparator and Genotype–Phenotype Classification

#### 2.3.1. Phenotypic AST Comparator and Binary Classification

Phenotypic antimicrobial susceptibility testing (AST) was performed at the central reference laboratory on reportable bacterial isolates recovered from quantitative urine culture. The parent-study dataset retained the original isolate-level minimum inhibitory concentration (MIC) results, where available, together with the prospectively assigned susceptible (S), intermediate (I), or resistant (R) interpretations.

For this secondary analysis, the original categorical AST interpretations were used without retrospective reinterpretation of MIC values or application of a new breakpoint standard. Where the archived parent-study documentation did not allow reliable reconstruction of the exact AST platform, antimicrobial-specific breakpoint, or breakpoint version for an individual comparison, those details were not inferred.

For binary genotype–phenotype agreement analyses, intermediate and resistant classifications were grouped as phenotypically non-susceptible and classified as AST-positive (I/R), whereas susceptible results were classified as AST-negative (S), consistent with the previously published parent-study AMR analysis [9]. The terms AST-positive and AST-negative therefore refer to the phenotypic comparator classification.

#### 2.3.2. Molecular AMR Target–Phenotype Mapping

Molecular AMR target results were compared with the corresponding phenotypic AST classifications generated during the parent clinical study rather than with independent molecular confirmation of individual resistance genes [5,6,7,8,9,10]. Each molecular AMR target or reporting group was evaluated against the applicable organism–antimicrobial phenotype used in the parent-study analytical framework. The organism(s), phenotypic AST comparator, resistance mechanism represented, and molecular/phenotypic classification rule for each target or reporting group are summarized in Supplementary Table S1.

For grouped molecular targets, a molecular-positive classification was assigned when at least one reportable determinant within the predefined reporting group was detected. The corresponding AST-positive classification was based on the mapped phenotypic non-susceptibility result for the applicable organism–antimicrobial relationship. Molecular reporting groups were not assumed to be mutually exclusive; therefore, a specimen could contribute to more than one AMR reporting category when multiple determinants were detected.

In polymicrobial specimens, detection of an AMR determinant was interpreted at the specimen level and did not assign that determinant to a specific organism unless organism–gene linkage had been established. No independent sequencing or second molecular assay was used to confirm individual AMR targets. The resulting comparisons therefore represent molecular AMR target–phenotypic AST agreement. Resistance mechanisms not included in the panel could contribute to phenotypic non-susceptibility, and where no appropriate phenotypic comparator was available, molecular detection alone was not used to assign genotype–phenotype agreement.

### 2.4. Framework for Evidence-Based Target Selection

#### 2.4.1. Evidence Review and Target-Prioritization Framework

To guide the transition from the V2 configuration to V5, existing and candidate targets were evaluated within the US clinical and epidemiological context across four domains: (1) epidemiological relevance, (2) antimicrobial-resistance relevance, (3) clinical and antimicrobial-stewardship actionability, and (4) species-level diagnostic and reporting value.

Existing and candidate targets were evaluated within the US intended-use context across four domains: epidemiological relevance, AMR relevance, clinical/antimicrobial-stewardship actionability, and species-level reporting value. Evidence was derived from a targeted review of surveillance data, professional guidance, and peer-reviewed literature, prioritizing contemporary US evidence relevant to adult cUTI/rUTI; the literature was updated through July 2026. The review was not systematic, and no single prevalence or resistance threshold determined target inclusion or exclusion. Target-level rationale and supporting references are provided in Supplementary Tables S2 and S3 [2,11,12,13,14,15,16,17,18,19,20,21,22,23,24,25,26,27,28,29,30,31,32,33,34,35,36,37].

#### 2.4.2. Classification of V2-to-V5 Target Modifications

For reporting purposes, V2-to-V5 target modifications were classified into seven operational categories (Table 2). The categories describe the type of target or reporting change and were used only to organize the reconfiguration framework.

The complete V2-to-V5 pathogen and AMR mappings, including the modification category, rationale, supporting evidence, and references associated with each target decision, are provided in Supplementary Tables S2 and S3, respectively.

### 2.5. Retrospective Archived-Specimen Assessment of the V5 Configuration

#### 2.5.1. Archived Specimen Selection and V5 Testing

**Figure 1:**
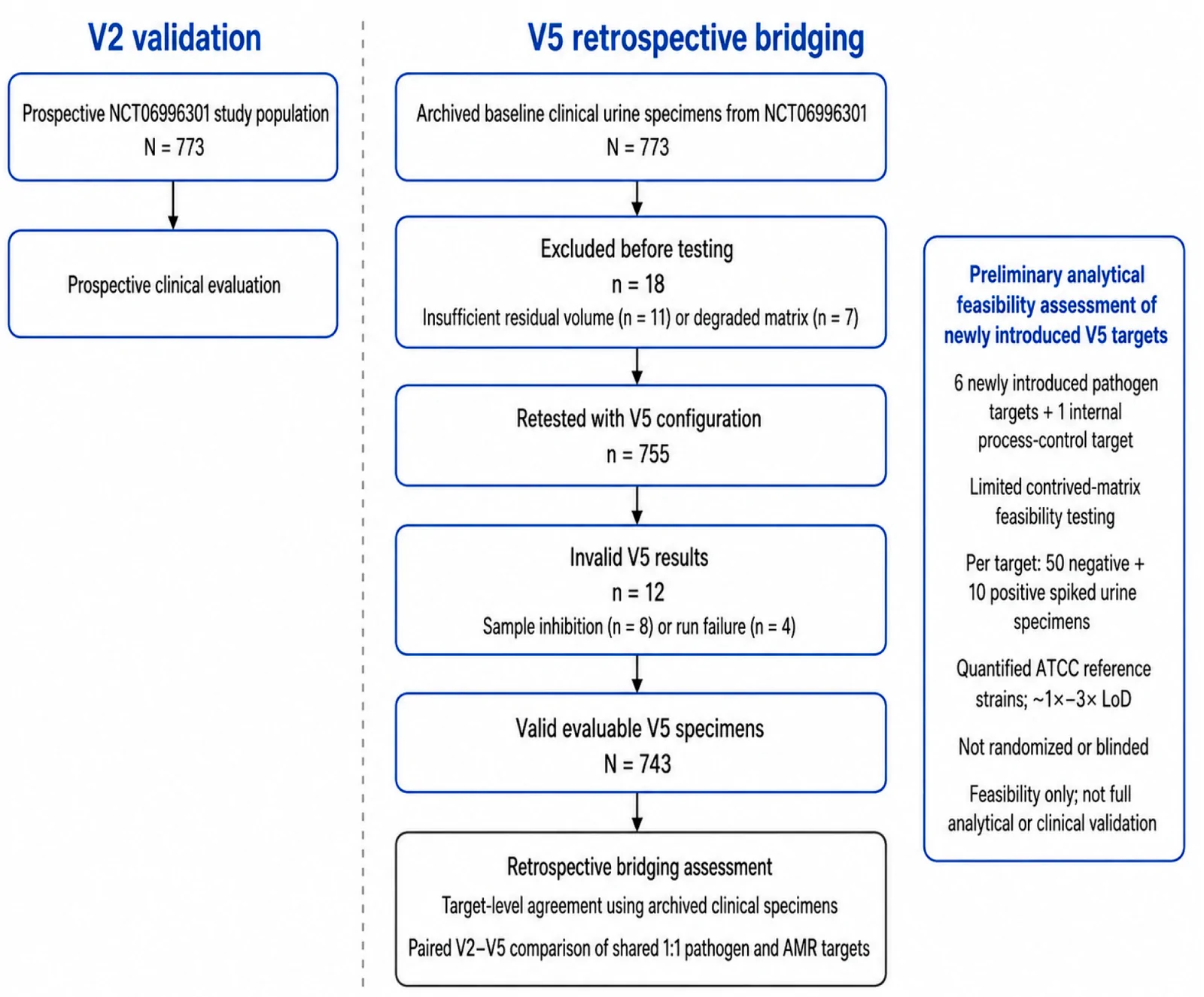
Study design and specimen flow.

A retrospective V5 assessment was performed using archived Visit 1 urine specimens from the prospective NCT06996301 study [5,6,7,8,9,10]. The archive initially comprised 773 baseline clinical specimens corresponding to the V2 analysis population. Eighteen specimens were excluded before V5 testing: 11 because of insufficient residual volume and 7 because of degraded matrix. The remaining 755 specimens were thawed and retested using the V5 configuration; 12 produced invalid V5 results, 8 because of sample inhibition and 4 because of run failure, resulting in 743 evaluable V5 specimens. This specimen flow is summarized in Figure 1 and Table 1.

Specimens were aliquoted following collection and stored in preservative-free sterile cryovials at −70 °C or below. The original quantitative urine culture and phenotypic AST results generated during the parent study were retained as the reference comparators. No new culture or AST testing was performed for the retrospective V5 assessment.

Archived specimens were thawed at room temperature and retested using the V5 configuration according to the applicable assay procedure, using the same nucleic-acid extraction and real-time PCR platforms described in Section 2.1. V5 pathogen and AMR targets were interpreted using the same qualitative reporting ranges applied in V2 (Cq 10–32 for pathogen targets and Cq 10–31 for AMR targets).

Only specimens with valid V5 results were included in the V5 agreement analyses.

#### 2.5.2. Paired V2–V5 Comparison Cohort

For direct V2–V5 comparisons of shared 1:1 pathogen and AMR targets, analyses were restricted to the 743 specimens with valid V2 and V5 molecular results and evaluable reference-comparator data for the directly shared targets. The same 743-specimen cohort was therefore used for all direct cross-version comparisons. Targets whose reporting definitions changed through unbundling, expansion, or consolidation were evaluated descriptively and were not treated as direct 1:1 paired comparisons.

The archived V5 analysis evaluated agreement with the original parent-study comparators and cross-version continuity for directly shared targets. It was not designed as a prospective clinical study or as comprehensive analytical validation of the final V5 configuration.

#### 2.5.3. Preliminary Analytical Feasibility Assessment of Newly Introduced Targets

Six newly introduced pathogen targets and the *Bacillus atrophaeus* internal process control were evaluated using contrived urine specimens. For each target, 10 positive and 50 negative specimens were tested. The negative matrix consisted of pooled human urine confirmed negative for applicable panel targets; positive specimens used quantified ATCC reference material prepared, where applicable, at approximately 1×–3× the developmental LoD estimate. No additional strains or clinical isolates were evaluated. The experiment was not randomized or blinded, and the laboratory personnel knew the specimen status. Testing was intended to assess target detectability and did not evaluate comprehensive inclusivity, exclusivity, interference, precision, reproducibility, or clinical performance.

### 2.6. Statistical Analysis

#### 2.6.1. Target-Level Agreement with Reference Comparators

Diagnostic agreement between molecular results and the available reference comparator was evaluated at the target level. For the V2 clinical dataset, and for V5 target analyses for which an appropriate clinical comparator was available, positive percent agreement (PPA), negative percent agreement (NPA), and overall percent agreement (OPA) were calculated from target-level 2 × 2 tables as TP/(TP+FN), TN/(TN+FP), and (TP+TN)/N, respectively, with two-sided Wilson 95% CIs.

Quantitative urine culture was the pathogen comparator and the mapped parent-study phenotypic AST classification was the AMR comparator (Supplementary Table S1). Intermediate/resistant results were classified as AST-positive/non-susceptible and susceptible results as AST-negative.

Invalid or non-evaluable results were excluded from the applicable denominator. Cohen’s κ was reported descriptively and as not estimable when the marginal distribution, including absence of reference-positive specimens, precluded meaningful estimation. These statistics were interpreted as agreement measures rather than absolute clinical sensitivity/specificity or independent molecular confirmation.

#### 2.6.2. Paired V2–V5 Comparisons and Multiplicity Adjustment

For direct V2–V5 comparisons, analyses were restricted to the common cohort of 743 specimens with valid V2 and V5 molecular results and evaluable comparator data for all directly shared 1:1 pathogen and AMR targets. Targets whose reporting definitions changed through unbundling, expansion, or consolidation were not included in formal 1:1 paired testing.

McNemar’s test was used to assess whether the frequency of discordant V2/V5 paired classifications differed between assay configurations. Testing was two-sided with α = 0.05; the exact McNemar test was used when discordant-pair counts were small.

Because multiple target-specific McNemar tests were performed, Holm adjustment was applied separately to the pathogen and AMR comparison families to control the family-wise error rate. Statistical inference was based on the multiplicity-adjusted *p*-values. Agreement estimates and their confidence intervals were descriptive and were not multiplicity-adjusted.

McNemar testing assessed directional differences in paired classifications. No equivalence margin was prespecified, and the retrospective V5 assessment was not prospectively powered for equivalence or non-inferiority.

#### 2.6.3. Contrived-Matrix Feasibility Analysis

For the contrived-matrix experiments, observed positive detection agreement, negative agreement, and overall agreement were summarized descriptively. These measures were used as feasibility outcomes and not as clinical performance estimates.

All statistical analyses were performed using R Statistical Software (version 4.2.2; R Foundation for Statistical Computing, Vienna, Austria).

## 3. Results

### 3.1. Baseline V2 Panel Target-Level Agreement

#### 3.1.1. V2 Pathogen-Level Agreement

Target-level agreement for the baseline V2 pathogen panel is summarized in Table 3. Among bacterial targets with at least one culture-positive comparator specimen, PPA estimates were consistently high, ranging from 96.7% to 100.0%. NPA showed greater between-target variation, with the lowest estimates observed for the composite *Klebsiella pneumoniae/oxytoca* (84.1%) and *Enterococcus faecium/faecalis* (86.2%) targets. The *Proteus mirabilis/vulgaris* composite target, in contrast, demonstrated high positive and negative agreement (PPA, 97.9%; NPA, 98.7%).

Several low-prevalence targets yielded 100% observed PPA but were represented by few culture-positive specimens and therefore had wide confidence intervals. These included *Acinetobacter baumannii* (4/4), *Streptococcus pyogenes* (7/7), *Ureaplasma urealyticum* (2/2), *Candida* spp. (4/4), and *Gardnerella vaginalis* (3/3). The corresponding confidence intervals reflect the limited precision of these estimates. No culture-positive specimens were identified for *Mycoplasma genitalium*, *Neisseria gonorrhoeae*, *Trichomonas vaginalis*, or *Chlamydia trachomatis*. Consequently, PPA and Cohen’s κ were not estimable for these targets; NPA estimates ranged from 97.3% to 98.3% (Table 3).

#### 3.1.2. V2 Molecular AMR Target–Phenotypic AST Agreement

The V2 AMR results are summarized in Table 4. Across molecular AMR targets with available positive phenotypic comparators, PPA estimates ranged from 97.1% to 100.0%, while NPA estimates ranged from 95.2% to 99.6%. Among the more frequently represented targets, CTX-M Group 1, *bla*KPC, *bla*NDM, *dfrA1/dfrA5/sul1/sul2*, *qnrB*, and *qnrS* demonstrated PPAs of 98.3–98.7% and NPAs of 95.2–97.6%.

The observed PPA was 100% for OXA-48, OXA-51, *mecA*, the *blaIMP/blaNDM/blaVIM* reporting group, and *qnrA1/qnrA2*. These estimates were based on relatively small numbers of phenotypically positive comparator specimens and therefore had wider confidence intervals and lower precision. For OXA-51 specifically, *blaOXA-51-like* genes are intrinsic to *A. baumannii*. The reported agreement reflects concordance with the mapped parent-study carbapenem phenotype and does not indicate that OXA-51 alone predicts carbapenem resistance.

As defined in Section 2.3 and Supplementary Table S1, these analyses compare molecular AMR-target detection with the corresponding phenotypic AST classifications. Molecular reporting groups were analyzed according to their predefined V2 reporting definitions and were not assumed to be mutually exclusive.

### 3.2. Panel Reconfiguration from V2 to V5 and Target-Level Rationale

#### 3.2.1. Pathogen Level Target Reconfiguration (V2 to V5)

The V2-to-V5 pathogen composition changes are summarized in Supplementary Table S2. Changes were classified according to the predefined categories described in Section 2.4, with the principal pathogen-level modifications comprising species-level unbundling, expansion of selected reporting categories, addition of selected targets, omission of selected targets, and retention of existing targets.

Several composite V2 targets were separated into species-level outputs in V5. *Enterococcus faecium/faecalis* was separated into *E. faecium* and *E. faecalis*; *Klebsiella pneumoniae/oxytoca* into *K. pneumoniae* and *K. oxytoca*; and *Proteus mirabilis/vulgaris* into *P. mirabilis* and *P. vulgaris*. The V2 *Staphylococcus* CoNS reporting category was expanded to include species-level outputs for *S. epidermidis*, *S. haemolyticus*, and *S. lugdunensis*. Similarly, the broad *Candida* spp. category was expanded to species-level reporting for *C. albicans*, *C. glabrata*, *C. parapsilosis*, and *C. tropicalis*. These changes increased species-level reporting granularity.

The V5 configuration also incorporated six additional pathogen targets not present in V2: *Providencia stuartii*, *Morganella morganii*, *Corynebacterium urealyticum*, the combined *Aerococcus urinae/A. sanguinicola* target, *Enterobacter cloacae*, and *Klebsiella aerogenes*. *Bacillus atrophaeus* was added separately as an internal molecular process-control target and was not considered a clinical pathogen target.

Selected V2 targets, including *Gardnerella vaginalis*, *Neisseria gonorrhoeae*, *Chlamydia trachomatis*, and *Trichomonas vaginalis*, were omitted from V5 as part of intended-use and multiplex-capacity prioritization for the cUTI/rUTI application. *Mycoplasma genitalium* and *Ureaplasma urealyticum* were retained as selected urogenital targets for context-dependent interpretation and were not classified as conventional cUTI pathogens.

Overall, the V5 pathogen architecture differed from V2 primarily in target composition and species-level reporting resolution. The complete V2-to-V5 pathogen mapping, modification category, scientific rationale, and supporting references are provided in Supplementary Table S2.

#### 3.2.2. AMR Target Reconfiguration (V2 to V5)

The V2-to-V5 AMR architecture is summarized in Supplementary Table S3. The principal substantive modification involved the plasmid-mediated quinolone-resistance targets, while the remaining major AMR targets and reporting groups were retained across configurations, with nomenclature or reporting standardization where applicable.

In V2, *qnrS* and *qnrA1/qnrA2* were reported separately, with *qnrB* included as an additional target. In V5, *qnrA* and *qnrS* were consolidated into a combined *qnrA/qnrS* reporting channel, while *qnrB* was omitted. This change reflected the fixed multiplex/reporting capacity of the redesigned panel. Combining *qnrA* and *qnrS* allowed two plasmid-mediated quinolone-resistance target families to be represented within one reporting channel, whereas retaining *qnrB* would have required an additional dedicated channel. The combined *qnrA/qnrS* channel was therefore prioritized as an assay-architecture decision; this was not based on inadequate V2 performance or lower biological relevance of *qnrB*. Consequently, V5 does not provide a dedicated *qnrB* result and does not retain identical qnr-target coverage to V2.

The other major AMR targets/reporting groups were retained across V2 and V5, with apparent differences in selected labels reflecting nomenclature standardization rather than changes in the underlying resistance targets. OXA-51 was retained from V2 within the existing AMR reporting architecture. Because *blaOXA-51-like* genes are intrinsic to *A. baumannii*, detection of this target alone was not interpreted as evidence of an acquired carbapenemase or as a standalone predictor of carbapenem resistance.

The complete V2-to-V5 AMR mapping and target-level rationale are provided in Supplementary Table S3; the separate molecular AMR target–phenotypic AST mapping used for the agreement analyses is provided in Supplementary Table S1.

### 3.3. Retrospective Clinical-Specimen Bridging Assessment of V5

From an initial cohort of 773 archived baseline urine specimens from NCT06996301, 18 were excluded before V5 testing: 11 because of insufficient residual volume and 7 because of degraded matrix. Of the remaining 755 specimens tested, 12 produced invalid V5 results (8 because of sample inhibition and 4 because of run failure), leaving 743 evaluable specimens (Table 1 and Figure 1). V5 pathogen and AMR results were evaluated against the corresponding culture and phenotypic AST results generated during the parent study.

The V5 assessment included target-level agreement against the archived clinical comparators, preliminary contrived-matrix feasibility testing of newly introduced targets, paired V2–V5 analysis of directly shared targets, and descriptive evaluation of species-level reporting following unbundling of composite V2 targets.

#### 3.3.1. V5 Pathogen-Level Diagnostic Agreement

Target-level agreement for the V5 configuration against the archived parent-study culture comparator is presented in Table 5. Among the more frequently represented established pathogen targets, PPA estimates ranged from 94.8% to 96.9%, while NPA estimates ranged from 87.0% to 98.5%. The lowest NPA among these targets was observed for *Klebsiella pneumoniae* (87.0%), followed by *Enterococcus faecalis* (90.3%). For *Escherichia coli* and *Pseudomonas aeruginosa*, PPA was 96.6% and 95.9%, respectively, while *K. pneumoniae* showed a PPA of 95.8%.

Several targets had few culture-positive comparator specimens and consequently imprecise PPA estimates. *Acinetobacter baumannii*, *Streptococcus pyogenes*, *Proteus vulgaris*, *Staphylococcus haemolyticus*, and *S. lugdunensis* showed 100% observed PPA, with wide confidence intervals reflecting the small positive denominators. *Ureaplasma urealyticum* had two culture-positive comparators and an observed PPA of 100% (95% CI, 34.2–100.0%) with an NPA of 93.4%.

No culture-positive specimens were available for *Mycoplasma genitalium* or *Candida parapsilosis*; therefore, PPA and Cohen’s κ were not estimable for these targets.

#### 3.3.2. Preliminary Analytical Feasibility Assessment of Newly Introduced V5 Targets

Six newly introduced pathogen targets and one internal molecular process-control target (7 total) were evaluated using contrived urine specimens. For each target, 60 specimens were tested, comprising 10 spiked-positive and 50 negative-matrix specimens. All 10 spiked-positive specimens were detected and all 50 negative specimens remained negative for each tested target (Table 6). These results demonstrate target detectability under the predefined contrived-matrix conditions.

**Table 6:** Preliminary Analytical Feasibility Assessment of Newly Introduced V5 Targets in Contrived Urine Matrix.

Target/ Process Control	Sample Type	Spiked Positives (*n*)	Clean Negatives (*n*)	Observed Positive Detection Agreement (95% CI)	Observed Negative Agreement (95% CI)	Observed Overall Agreement (95% CI)
*Providencia stuartii*	Spiked Urine	10	50	100.0% [72.2–100.0]	100.0% [92.9–100.0]	100.0% [94.0–100.0]
*Morganella morganii*	Spiked Urine	10	50	100.0% [72.2–100.0]	100.0% [92.9–100.0]	100.0% [94.0–100.0]
*Enterobacter cloacae*	Spiked Urine	10	50	100.0% [72.2–100.0]	100.0% [92.9–100.0]	100.0% [94.0–100.0]
*Klebsiella aerogenes*	Spiked Urine	10	50	100.0% [72.2–100.0]	100.0% [92.9–100.0]	100.0% [94.0–100.0]
*Corynebacterium urealyticum*	Spiked Urine	10	50	100.0% [72.2–100.0]	100.0% [92.9–100.0]	100.0% [94.0–100.0]
*Aerococcus urinae/A. sanguinicola * *(combined target)*	Spiked Urine	10	50	100.0% [72.2–100.0]	100.0% [92.9–100.0]	100.0% [94.0–100.0]
*Bacillus atrophaeus * *(internal process control)*	Spiked Urine	10	50	100.0% [72.2–100.0]	100.0% [92.9–100.0]	100.0% [94.0–100.0]

Testing used limited reference-strain material and was intended as a preliminary analytical feasibility assessment only. The experiment was not randomized or blinded. These data do not constitute comprehensive analytical validation or clinical performance estimates.

#### 3.3.3. Paired V2–V5 Comparison of Shared Pathogen Targets

**Figure 2:**
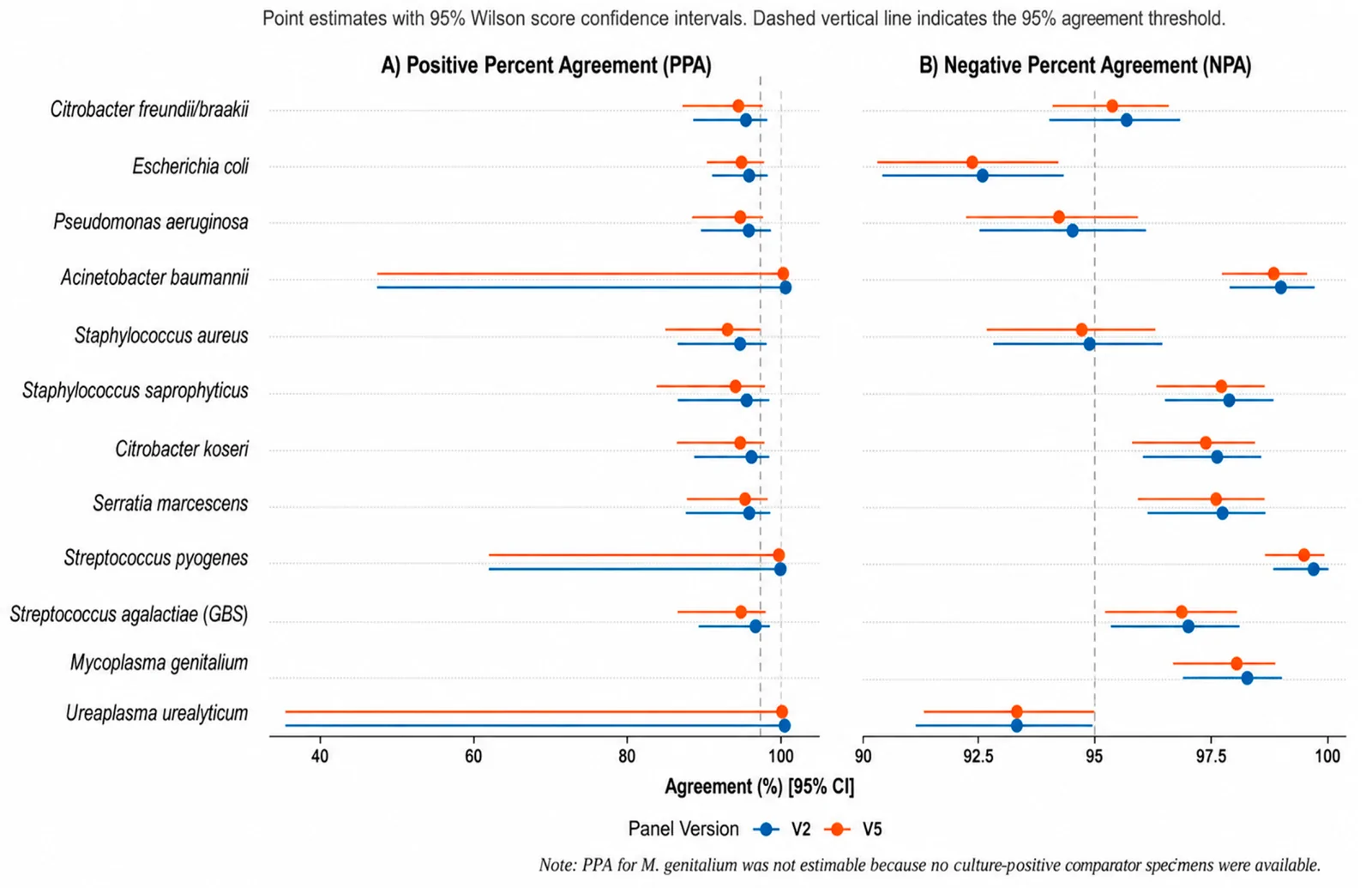
Paired Comparison of Shared Pathogen Targets between V2 and V5 (N = 743).

Direct comparisons of shared 1:1 pathogen targets were restricted to the common cohort of 743 specimens with valid V2 and V5 results and evaluable culture-comparator data. V2 and V5 classifications for each shared pathogen target were compared within the same specimens, thereby preserving the paired structure of the analysis. Corresponding target-level agreement with the culture comparator is presented for V2 and V5 in Table 3 and Table 5, respectively, and summarized graphically in Figure 2.

PPA and NPA estimates for the directly shared targets were descriptively similar across V2 and V5, with no consistent directional pattern of change. After Holm adjustment for multiple target-specific McNemar comparisons, no statistically significant differences in paired molecular classifications were observed among the directly shared pathogen targets (all adjusted *p* > 0.05).

The paired analysis was not designed to test equivalence or non-inferiority, and no equivalence margin was prespecified.

#### 3.3.4. Species-Level Reporting Resolution Following Target Unbundling

**Figure 3:**
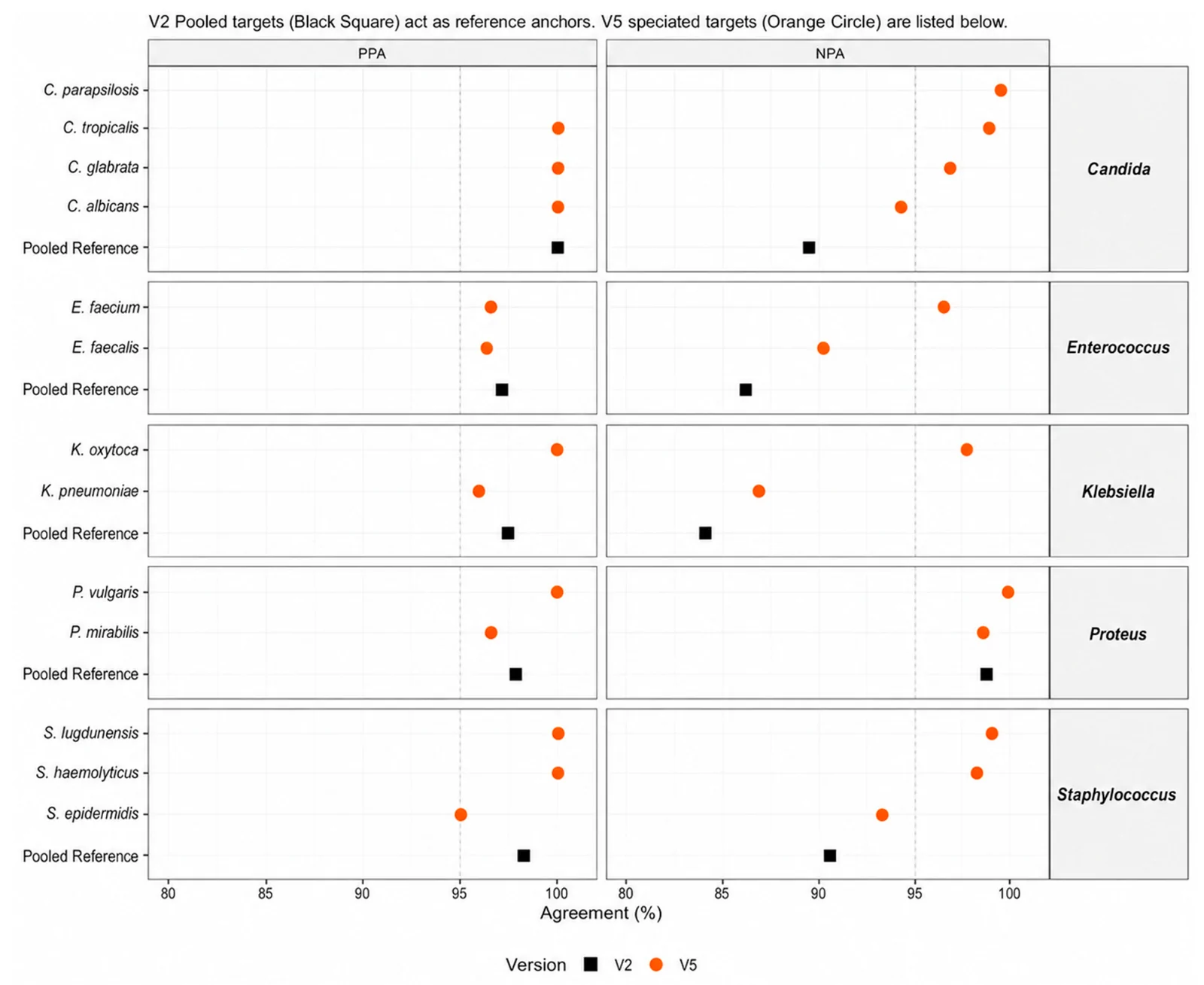
Reporting Resolution: V2 Composite Targets vs. V5 Species-Level Outputs.

The transition from composite V2 reporting categories to species-resolved V5 targets increased reporting granularity by partitioning organism detection and associated discordances across species-specific reporting channels (Figure 3). Because the composite and species-level targets have different reporting definitions, these comparisons were descriptive and were not analyzed as direct 1:1 paired target comparisons.

The V2 composite *Enterococcus faecium/faecalis* target had a PPA of 97.2% and NPA of 86.2%. In V5, the corresponding species-specific targets yielded PPA/NPA estimates of 96.3%/90.3% for *E. faecalis* and 96.4%/96.5% for *E. faecium*. The observed discordances were consequently distributed across separate species-specific reporting channels.

Similarly, separation of the V2 *Klebsiella pneumoniae/oxytoca* and *Proteus mirabilis/vulgaris* composite targets generated independent species-specific estimates in V5. Together, these results document the increased species-level reporting resolution produced by target unbundling.

### 3.4. V5 Molecular AMR Target–Phenotypic AST Agreement

Molecular AMR target–phenotypic AST agreement for the V5 configuration is summarized in Table 7. Across the evaluated targets, PPA ranged from 94.9% to 100.0% and NPA from 96.6% to 99.6%. Most retained AMR targets showed high agreement with their corresponding parent-study phenotypic AST comparators. Several low-prevalence targets, including OXA-48, OXA-51, *mecA*, and the *blaIMP/blaNDM/blaVIM* reporting group, showed 100% observed PPA, with confidence intervals reflecting the small numbers of phenotypically positive comparator specimens.

The principal V5 AMR reporting change involved the *qnr* targets. The separate V2 *qnrA1/qnrA2* and *qnrS* reporting channels were consolidated into a combined *qnrA/qnrS* target, while *qnrB* was omitted, as described in Section 3.2.2. The combined V5 *qnrA/qnrS* target demonstrated a PPA of 95.7%, NPA of 96.8%, and Cohen’s κ of 0.769. These values describe agreement of the consolidated V5 target with its mapped phenotypic comparator. Because its reporting definition differs from the separate V2 *qnrA1/qnrA2* and *qnrS* outputs, it was not analyzed as a direct 1:1 paired target comparison.

As defined in Section 2.3 and Supplementary Table S1, the reported AMR estimates represent genotype–phenotype agreement with the corresponding parent-study AST classifications. For OXA-51, the estimate reflects agreement with the mapped carbapenem phenotype; because *blaOXA-51-like* genes are intrinsic to *A. baumannii*, detection of this target alone does not establish carbapenem resistance.

#### Paired V2–V5 Comparison of Shared AMR Targets

**Figure 4:**
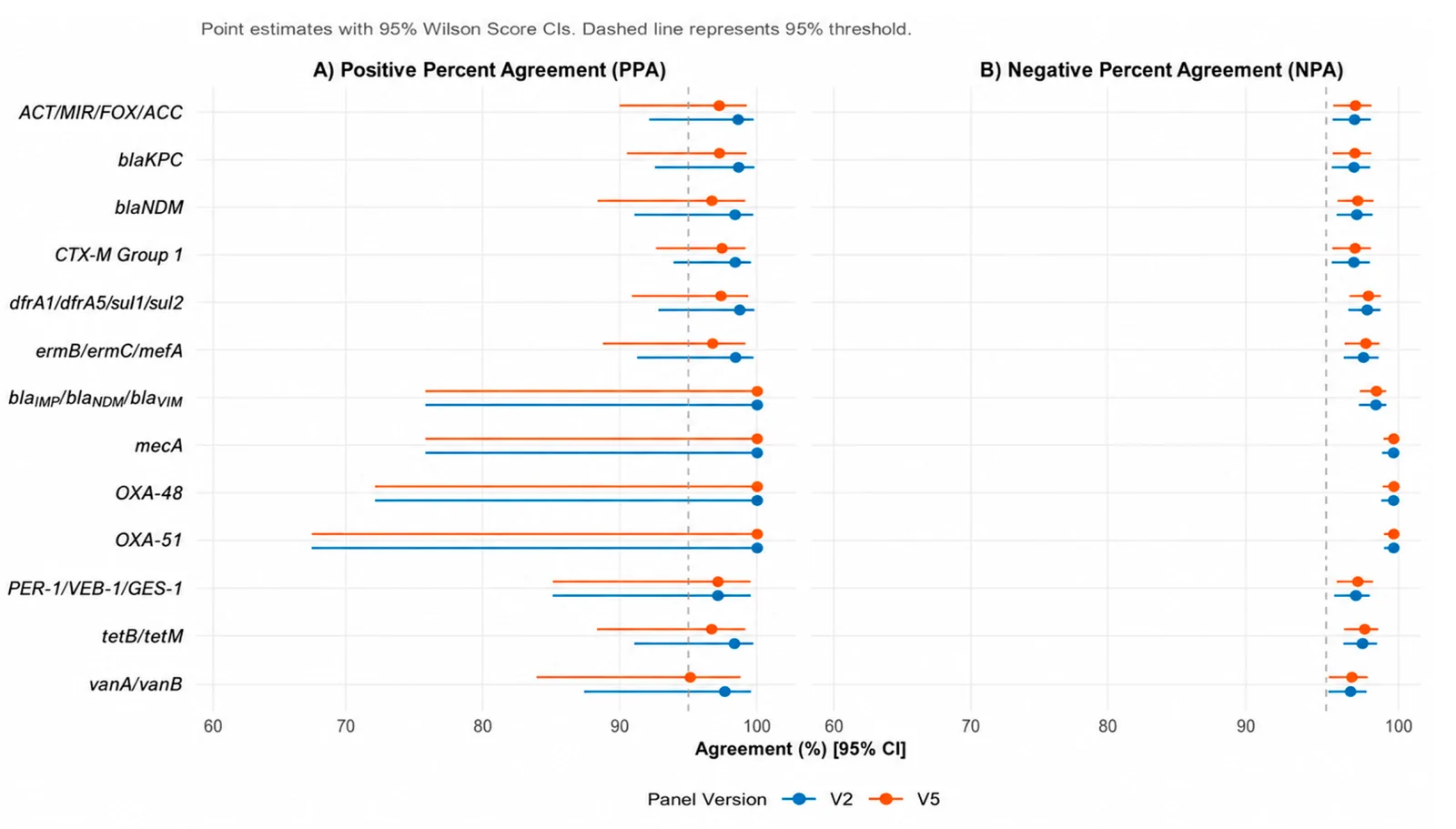
Paired Comparison of Shared Molecular AMR Targets between V2 and V5 (N = 743).

Direct comparison of shared AMR targets between V2 and V5 was restricted to the common cohort of 743 specimens with valid V2 and V5 molecular results and evaluable corresponding phenotypic AST data. Molecular classifications were compared within the same specimens for directly shared 1:1 AMR targets. V2 and V5 genotype–phenotype agreement estimates for this common cohort are summarized in Figure 4.

Across the directly shared AMR targets, PPA and NPA estimates against the corresponding parent-study phenotypic AST comparators were descriptively similar between V2 and V5, with no consistent directional pattern of change. Estimates for several low-prevalence targets remained imprecise because of the small numbers of phenotypically positive comparator specimens.

After Holm adjustment for multiple target-specific McNemar comparisons, no statistically significant differences in paired molecular classifications were observed among the directly shared AMR targets (all adjusted *p* > 0.05).

The analysis was not designed to test equivalence or non-inferiority, and no equivalence margin was prespecified.

## 4. Discussion

This study links the prospective V2 evidence base with retrospective assessment of the reconfigured V5 multiplex PCR panel. V5 was evaluated using archived specimens for pathogen agreement with quantitative culture, molecular AMR target–phenotypic AST agreement, and paired comparison of directly shared V2–V5 targets; newly introduced targets were assessed separately in contrived urine. Accordingly, the V5 findings constitute bridging evidence rather than independent prospective clinical validation.

### 4.1. Baseline V2 Pathogen and AMR Agreement

V2 showed high positive agreement for most established bacterial uropathogens, with greater variability among composite and low-prevalence targets. PCR–culture discordance is expected because culture depends on viable-organism recovery whereas PCR detects target nucleic acid; prior antimicrobial exposure, low organism burden, polymicrobial competition, fastidious growth, specimen handling, and quantitative thresholds may contribute [1,2,3,8]. Conversely, PCR may detect colonization, contamination, or residual nonviable nucleic acid. All participants were symptomatic with suspected cUTI, but symptoms do not establish that every detected organism was causative. Because discordant specimens were not independently adjudicated, individual discrepancies cannot be assigned to failure of either method. Estimates of 100% PPA for uncommon targets should likewise be interpreted with their wide confidence intervals.

The V2 AMR module likewise showed generally high agreement with the prespecified phenotypic AST comparators. These results describe genotype–phenotype agreement rather than direct confirmation of individual resistance genes. Gene detection and phenotypic susceptibility are related but not interchangeable: a detected determinant may not produce phenotypic non-susceptibility, while resistance can arise through mechanisms not represented in the molecular panel.

### 4.2. Panel Reconfiguration, Species-Level Reporting, and AMR Architecture

V2-to-V5 reconfiguration emphasized target composition and reporting resolution rather than simple panel expansion (Supplementary Tables S2 and S3). Unbundling composite *Enterococcus*, *Klebsiella*, *Proteus*, CoNS, and *Candida* reporting increased species-level resolution but did not eliminate PCR–culture discordance or establish improved analytical specificity or clinical outcomes.

The principal AMR change involved the *qnr* architecture. V2 *qnrS* and *qnrA1/qnrA2* were consolidated into a V5 *qnrA/qnrS* reporting channel, while *qnrB* was omitted. This reflected fixed multiplex capacity: consolidation retained two *qnr* target families within one channel, whereas *qnrB* required an additional channel. The decision was therefore an architectural trade-off, not evidence of poorer *qnrB* performance or lower biological relevance. OXA-51 was retained from V2; because *blaOXA-51-like* genes are intrinsic to *A. baumannii*, detection alone should not be interpreted as an acquired carbapenemase or standalone predictor of carbapenem resistance.

Less conventional urogenital targets also require intended-use context. *M. genitalium* is associated with urethritis rather than conventional cUTI, and *U. urealyticum* requires clinical correlation because colonization is common [38,39,40]. Omission of *G. vaginalis*, *N. gonorrhoeae*, *C. trachomatis*, and *T. vaginalis* reflects target prioritization rather than lack of clinical relevance. Because prioritization relied mainly on US evidence, other regions may require different target choices based on local epidemiology and resistance patterns.

### 4.3. V5 Retrospective Assessment and Cross-Version Bridging

Direct cross-version comparisons were restricted to the common 743-specimen cohort for directly shared 1:1 targets. After Holm adjustment, no statistically significant directional differences in paired pathogen or AMR classifications were identified (all adjusted *p* > 0.05). These findings support continuity of classification but do not establish equivalence or non-inferiority because no equivalence margin was prespecified and the study was not prospectively powered for those hypotheses.

For AMR targets, gene detection remains distinct from phenotypic susceptibility, and organism–gene linkage cannot always be assigned in polymicrobial specimens. The AMR findings should therefore be interpreted as genotype–phenotype agreement rather than organism-specific molecular susceptibility testing.

### 4.4. Newly Introduced V5 Targets

Newly introduced pathogen targets were supported only by preliminary contrived-matrix testing. The experiment demonstrated detectability under the tested conditions but used limited reference-strain material, was not randomized or blinded, and did not assess comprehensive inclusivity, exclusivity, interference, precision, reproducibility, or clinical agreement. Broader analytical and clinical validation is therefore required.

### 4.5. Clinical Implications, Strengths, and Limitations

The principal potential advantage of V5 is increased reporting resolution, which may reduce ambiguity associated with composite molecular signals. Whether the revised architecture improves antimicrobial selection, de-escalation, time to appropriate therapy, recurrence, hospitalization, or other outcomes was not evaluated; prospective V2 clinical utility findings therefore should be extrapolated to V5 with cautious.

Strengths include the well-characterized parent cohort, preservation of original culture and AST comparators, explicit specimen-flow accounting, use of a common paired cohort, and separation of archived clinical-specimen evidence from feasibility testing. Limitations include the retrospective use of archived specimens, exclusion or invalidity of 30 specimens, imperfect culture comparison without independent discordance adjudication, reliance on original AST classifications and predefined genotype–phenotype mappings rather than independent molecular confirmation, incomplete reconstruction of some AST platform/drug/breakpoint details, uncertain organism–gene attribution in polymicrobial specimens, small positive denominators for several targets, and limited feasibility testing of newly introduced targets.

Overall, the data support retrospective continuity for directly shared targets and increased species-level reporting resolution, but not superior analytical performance, improved stewardship, or better clinical outcomes. Comprehensive validation and prospective evaluation of the final V5 configuration remain required.

## 5. Conclusions

The V2-to-V5 DocLab UTM™ reconfiguration increased species-level reporting resolution and modified selected AMR reporting while retaining generally high retrospective agreement with the original parent-study culture and phenotypic AST comparators. In the common 743-specimen cohort, directly shared V2 and V5 pathogen and AMR targets showed no statistically significant directional differences in paired classifications after Holm adjustment; these findings support classification continuity and supports the plausibility that key clinical benefits observed with V2 may be preserved.

V5 was assessed retrospectively, and newly introduced targets were supported only by limited contrived-matrix feasibility testing. The present evidence therefore does not establish superior analytical specificity, improved stewardship, or improved clinical outcomes. Comprehensive analytical validation and prospective evaluation of the final V5 configuration remain necessary.

## Figures and Tables

**Table 1:** Participant and specimen eligibility and evaluability across the V2 and V5 analyses.

Stage	Inclusion/Evaluability Requirement	Exclusion/Non-Evaluability Criterion
Parent NCT06996301 cohort	Protocol-defined symptomatic adult cUTI eligibility; pyuria; treatment-failure or higher-risk criteria; ability to provide ≥8 mL urine [5]	Protocol-defined parent-study exclusion criteria; full criteria reported in Ref. [5]
V2 baseline analysis	Evaluable baseline V2 specimen with corresponding reference-comparator data	No additional retrospective exclusion beyond parent-study baseline evaluability
Archived V5 testing	Archived Visit 1 specimen available and suitable for V5 retesting	Insufficient residual volume (*n* = 11) or degraded matrix (*n* = 7)
V5 evaluable cohort	Valid V5 molecular result	Sample inhibition (*n* = 8) or run failure (*n* = 4)
Paired V2–V5 analysis	Valid V2 and V5 results within the common 743-specimen cohort for directly shared 1:1 targets	Targets whose reporting definitions changed through unbundling, expansion, or consolidation were excluded from direct 1:1 paired testing

**Table 2:** Operational categories used to classify V2-to-V5 target modifications.

Categories	Definition
Unchanged	Retained without alteration
Notation Update	Nomenclature or reporting revision without a substantive change in target coverage
Updated (Unbundled)	Separation of a composite target into individual species-level targets
Updated (Expanded)	Replacement of a broader grouping with additional species-specific targets
Omitted	Removal from the V5 configuration
New Addition	Incorporation of a target not present in V2
Consolidated	Combination of previously separate targets into a single reporting channel

**Table 3:** V2 Pathogen-Level Diagnostic Agreement in the NCT06996301 Baseline Cohort (N = 773).

Pathogen	PCR+	Urine Culture+	TP	TN	FP	FN	PPA (95% CI)	NPA (95% CI)	OPA (95% CI)	Cohen’s Kappa (κ)
*Citrobacter freundii/braakii*	73	43	42	699	31	1	97.6% (87.9–99.6%)	95.8% (94.0–97.0%)	95.9% (94.2–97.1%)	0.703
*Escherichia coli*	168	123	120	602	48	3	97.6% (93.1–99.2%)	92.6% (90.3–94.4%)	93.4% (91.4–95.0%)	0.785
*Pseudomonas aeruginosa*	134	101	98	636	36	3	97.0% (91.7–99.1%)	94.6% (92.7–96.1%)	95.0% (93.2–96.4%)	0.805
*Acinetobacter baumannii*	13	4	4	760	9	0	100.0% (51.0–100.0%)	98.8% (97.8–99.4%)	98.8% (97.8–99.4%)	0.466
*Staphylococcus aureus*	96	61	59	675	37	2	96.7% (88.5–99.6%)	94.8% (92.9–96.2%)	95.0% (93.2–96.4%)	0.725
*Staphylococcus saprophyticus*	62	46	45	710	17	1	97.8% (88.4–99.9%)	97.7% (96.3–98.6%)	97.7% (96.3–98.6%)	0.821
*Citrobacter koseri*	75	58	57	697	18	1	98.3% (90.6–99.8%)	97.5% (96.0–98.4%)	97.5% (96.2–98.4%)	0.844
*Serratia marcescens*	81	66	64	690	17	2	97.0% (89.2–99.5%)	97.6% (96.1–98.5%)	97.5% (96.2–98.4%)	0.857
*Streptococcus pyogenes*	11	7	7	762	4	0	100.0% (64.6–100.0%)	99.5% (98.7–99.8%)	99.5% (98.6–99.7%)	0.775
*Streptococcus agalactiae *(GBS)	78	57	56	694	22	1	98.2% (90.6–99.8%)	96.9% (95.4–98.1%)	97.0% (95.5–98.0%)	0.814
*Mycoplasma genitalium*	15	0	0	758	15	0	N/A	98.1% (96.8–98.8%)	98.1% (96.8–98.8%)	N/A
*Ureaplasma urealyticum*	54	2	2	719	52	0	100.0% (34.2–100.0%)	93.3% (91.2–94.9%)	93.3% (91.3–94.9%)	0.067
*Enterococcus faecium/faecalis*	197	108	105	573	92	3	97.2% (92.3–99.0%)	86.2% (83.3–88.7%)	87.7% (85.2–89.8%)	0.620
*Klebsiella pneumoniae/oxytoca*	219	118	115	551	104	3	97.5% (92.6–99.1%)	84.1% (81.1–86.8%)	86.2% (83.5–88.4%)	0.604
*Proteus mirabilis/vulgaris*	104	97	95	667	9	2	97.9% (92.1–99.7%)	98.7% (97.5–99.3%)	98.6% (97.5–99.2%)	0.937
*Staphylococcus *(CoNS)	124	58	57	648	67	1	98.3% (90.9–99.8%)	90.6% (88.1–92.6%)	91.2% (89.0–93.0%)	0.584
*Candida* spp.	85	4	4	688	81	0	100.0% (51.0–100.0%)	89.5% (87.2–91.5%)	89.5% (87.2–91.5%)	0.081
*Gardnerella vaginalis*	41	3	3	732	38	0	100.0% (63.1–100.0%)	95.1% (93.3–96.4%)	95.1% (93.3–96.4%)	0.130
*Neisseria gonorrhoeae*	19	0	0	754	19	0	N/A	97.5% (96.2–98.4%)	97.5% (96.2–98.4%)	N/A
*Trichomonas vaginalis*	21	0	0	752	21	0	N/A	97.3% (95.9–98.2%)	97.3% (95.9–98.2%)	N/A
*Chlamydia trachomatis*	13	0	0	760	13	0	N/A	98.3% (97.1–99.0%)	98.3% (97.1–99.0%)	N/A

TP, true positive; TN, true negative; FP, molecular-positive/culture-negative; FN, molecular-negative/culture-positive; PPA, positive percent agreement; NPA, negative percent agreement; OPA, overall percent agreement; CI, confidence interval; κ, Cohen’s kappa; CoNS, coagulase-negative staphylococci; N/A, not applicable/not estimable. PPA and κ were not estimated when no culture-positive comparator specimens were available. Agreement estimates describe concordance with quantitative urine culture and should not be interpreted as absolute clinical sensitivity or specificity.

**Table 4:** V2 Molecular AMR Target–Phenotypic AST Agreement in the NCT06996301 Baseline Cohort (N = 773).

Molecular AMR Target/Reporting Group	PCR+	AST+	TP	TN	FP	FN	PPA% [95% CI]	NPA% [95% CI]	OPA% [95% CI]	Cohen’s Kappa (κ)
* CTX-M Group 1 *	136	115	113	635	23	2	98.3% [93.9–99.5%]	96.5% [94.8–97.7%]	96.8% [95.3–97.8%]	0.875
* blaKPC *	95	72	71	677	24	1	98.6% [92.5–99.8%]	96.6% [94.9–97.7%]	96.8% [95.3–97.8%]	0.832
* blaNDM *	81	59	58	691	23	1	98.3% [91.0–99.7%]	96.8% [95.2–97.9%]	96.9% [95.4–97.9%]	0.815
* OXA-48 *	14	10	10	759	4	0	100.0% [72.2–100.0%]	99.5% [98.6–99.8%]	99.5% [98.7–99.8%]	0.831
* OXA-51 *	11	8	8	762	3	0	100.0% [67.6–100.0%]	99.6% [98.9–99.9%]	99.6% [98.9–99.9%]	0.840
* tetB/tetM *	77	59	58	695	19	1	98.3% [91.0–99.7%]	97.3% [95.9–98.3%]	97.4% [96.0–98.3%]	0.840
* ACT/MIR/FOX/ACC *	89	66	65	683	24	1	98.5% [91.9–99.7%]	96.6% [95.0–97.7%]	96.8% [95.3–97.8%]	0.820
* PER-1/VEB-1/GES-1 *	57	34	33	715	24	1	97.1% [85.1–99.5%]	96.8% [95.2–97.8%]	96.8% [95.3–97.8%]	0.706
* vanA/vanB *	66	41	40	706	26	1	97.6% [87.4–99.6%]	96.4% [94.8–97.6%]	96.5% [94.9–97.6%]	0.726
* dfrA1/dfrA5/sul1/sul2 *	91	75	74	681	17	1	98.7% [92.8–99.8%]	97.6% [96.1–98.5%]	97.7% [96.3–98.5%]	0.878
* ermB/ermC/mefA *	79	61	60	693	19	1	98.4% [91.2–99.7%]	97.3% [95.8–98.3%]	97.4% [96.0–98.3%]	0.844
* mecA *	16	12	12	757	4	0	100.0% [75.8–100.0%]	99.5% [98.7–99.8%]	99.5% [98.7–99.8%]	0.855
* blaIMP/blaNDM/blaVIM group *	26	12	12	747	14	0	100.0% [75.8–100.0%]	98.2% [97.0–99.0%]	98.2% [97.0–99.0%]	0.624
* qnrB *	94	61	60	678	34	1	98.4% [91.2–99.7%]	95.2% [93.4–96.6%]	95.5% [93.8–96.7%]	0.748
* qnrS *	85	63	62	687	23	1	98.4% [91.5–99.7%]	96.8% [95.1–97.9%]	96.9% [95.4–97.9%]	0.822
* qnrA1/qnrA2 *	12	9	9	761	3	0	100.0% (70.1–100.0%)	99.6% (98.9–99.9%)	99.6% (98.9–99.9%)	0.855

AST+, phenotypically non-susceptible (intermediate or resistant [I/R]) according to the applicable parent-study organism–antimicrobial mapping; AST−, phenotypically susceptible (S); TP, true positive; TN, true negative; FP, molecular-positive/AST-negative; FN, molecular-negative/AST-positive; PPA, positive percent agreement; NPA, negative percent agreement; OPA, overall percent agreement; CI, confidence interval; κ, Cohen’s kappa; AMR, antimicrobial resistance. Molecular AMR target–phenotypic AST mappings and interpretation rules are provided in Supplementary Table S1. Molecular reporting groups were not necessarily mutually exclusive. The estimates represent genotype–phenotype agreement with the parent-study phenotypic AST classifications and not independent molecular confirmation of individual resistance genes. Molecular detection of a resistance determinant does not itself establish phenotypic non-susceptibility, and absence of a targeted determinant does not establish phenotypic susceptibility. OXA-51 was retained within the assay AMR reporting architecture as an A. baumannii-associated oxacillinase target. Because blaOXA-51-like genes are intrinsic to A. baumannii, detection of this target alone should not be interpreted as evidence of carbapenem resistance or as an independent predictor of the carbapenem phenotype.

**Table 5:** V5 Pathogen-Level Diagnostic Agreement Using Archived Clinical Specimens (N = 743 evaluable of 773 archived specimens).

Pathogen/Target	PCR+	Urine Culture+	TP	TN	FP	FN	PPA% [95% CI]	NPA% [95% CI]	OPA% [95% CI]	Cohen’s Kappa (κ)
*Citrobacter freundii/braakii*	69	41	39	672	30	2	95.1% [83.9–98.7]	95.7% [94.0–97.0]	95.7% [94.0–97.0]	0.684
*Escherichia coli*	162	119	115	577	47	4	96.6% [91.6–98.7]	92.5% [90.1–94.3]	93.1% [91.1–94.8]	0.778
*Pseudomonas aeruginosa*	129	97	93	610	36	4	95.9% [89.9–98.4]	94.4% [92.4–95.9]	94.6% [92.8–96.1]	0.792
*Acinetobacter baumannii*	14	4	4	729	10	0	100.0% [51.0–100.0]	98.6% [97.5–99.3]	98.7% [97.6–99.3]	0.438
*Staphylococcus aureus*	91	58	55	649	36	3	94.8% [85.9–98.2]	94.7% [92.8–96.2]	94.7% [92.9–96.1]	0.704
*Staphylococcus saprophyticus*	59	44	42	682	17	2	95.5% [84.9–98.7]	97.6% [96.2–98.5]	97.4% [96.0–98.4]	0.798
*Citrobacter koseri*	72	56	54	669	18	2	96.4% [87.7–99.0]	97.4% [95.9–98.3]	97.3% [95.9–98.3]	0.825
*Serratia marcescens*	79	64	62	662	17	2	96.9% [89.3–99.1]	97.5% [96.0–98.5]	97.4% [96.0–98.4]	0.849
*Streptococcus pyogenes*	12	7	7	731	5	0	100.0% [64.6–100.0]	99.3% [98.4–99.7]	99.3% [98.4–99.7]	0.733
*Streptococcus agalactiae*	75	55	53	666	22	2	96.4% [87.7–99.0]	96.8% [95.2–97.9]	96.8% [95.3–97.9]	0.794
*Mycoplasma genitalium*	15	0	0	728	15	0	N/A	98.0% [96.7–98.8]	98.0% [96.7–98.8]	N/A
*Ureaplasma urealyticum*	51	2	2	692	49	0	100.0% [34.2–100.0]	93.4% [91.3–95.0]	93.4% [91.3–95.0]	0.068
*Enterococcus faecalis*	141	80	77	599	64	3	96.3% [89.6–98.7]	90.3% [87.8–92.4]	91.0% [88.7–92.9]	0.651
*Enterococcus faecium*	52	28	27	690	25	1	96.4% [82.3–99.4]	96.5% [94.9–97.6]	96.5% [94.9–97.6]	0.653
*Klebsiella pneumoniae*	176	96	92	563	84	4	95.8% [89.8–98.4]	87.0% [84.2–89.4]	88.2% [85.6–90.3]	0.610
*Klebsiella oxytoca*	34	18	18	709	16	0	100.0% [81.5–100.0]	97.8% [96.4–98.6]	97.8% [96.5–98.7]	0.680
*Proteus mirabilis*	91	84	81	649	10	3	96.4% [90.1–98.8]	98.5% [97.2–99.2]	98.2% [97.0–99.0]	0.915
*Proteus vulgaris*	12	11	11	731	1	0	100.0% [74.1–100.0]	99.9% [99.2–100.0]	99.9% [99.2–100.0]	0.956
*Staphylococcus epidermidis*	84	39	37	657	47	2	94.9% [83.1–98.6]	93.3% [91.2–95.0]	93.4% [91.4–95.0]	0.568
*Staphylococcus haemolyticus*	25	12	12	718	13	0	100.0% [75.8–100.0]	98.2% [97.0–98.9]	98.2% [97.0–98.9]	0.641
*Staphylococcus lugdunensis*	13	5	5	730	8	0	100.0% [56.6–100.0]	98.9% [97.9–99.4]	98.9% [97.9–99.4]	0.550
*Candida albicans*	45	3	3	698	42	0	100.0% [43.9–100.0]	94.3% [92.4–95.8]	94.3% [92.4–95.8]	0.117
*Candida glabrata*	25	1	1	718	24	0	100.0% [20.7–100.0]	96.8% [95.2–97.9]	96.8% [95.2–97.9]	0.073
*Candida tropicalis*	10	1	1	733	9	0	100.0% [20.7–100.0]	98.8% [97.7–99.4]	98.8% [97.7–99.4]	0.178
*Candida parapsilosis*	5	0	0	738	5	0	N/A	99.3% [98.4–99.7]	99.3% [98.4–99.7]	N/A

TP, true positive; TN, true negative; FP, molecular-positive/culture-negative; FN, molecular-negative/culture-positive; PPA, positive percent agreement; NPA, negative percent agreement; OPA, overall percent agreement; CI, confidence interval; κ, Cohen’s kappa; CoNS, coagulase-negative staphylococci; N/A, not applicable/not estimable. PPA and κ were not estimated when no culture-positive comparator specimens were available. Agreement estimates describe concordance with quantitative urine culture and should not be interpreted as absolute clinical sensitivity or specificity.

**Table 7:** V5 Molecular AMR Target–Phenotypic AST Agreement (N = 743 evaluable of 773 archived specimens).

Molecular AMR Target/Reporting Group	PCR+	AST+	TP	TN	FP	FN	PPA% [95% CI]	NPA% [95% CI]	OPA% [95% CI]	Cohen’s Kappa (κ)
* CTX-M Group 1 *	129	111	108	611	21	3	97.3% [92.3–99.1]	96.7% [95.0–97.8]	96.8% [95.2–97.8]	0.871
* blaKPC *	89	69	67	652	22	2	97.1% [90.2–99.2]	96.7% [95.1–97.9]	96.8% [95.2–97.9]	0.825
* blaNDM *	76	57	55	665	21	2	96.5% [88.1–99.1]	96.9% [95.4–98.0]	96.9% [95.4–98.0]	0.806
* OXA-48 *	13	10	10	730	3	0	100.0% [72.2–100.0]	99.6% [98.8–99.9]	99.6% [98.8–99.9]	0.867
* OXA-51 *	11	8	8	732	3	0	100.0% [67.6–100.0]	99.6% [98.8–99.9]	99.6% [98.8–99.9]	0.840
* tetB/tetM *	72	57	55	669	17	2	96.5% [88.1–99.1]	97.5% [96.0–98.5]	97.4% [96.0–98.4]	0.835
* ACT/MIR/FOX/ACC *	84	64	62	657	22	2	96.9% [89.3–99.1]	96.8% [95.2–97.9]	96.8% [95.3–97.8]	0.816
* PER-1/VEB-1/GES-1 *	53	33	32	689	21	1	97.0% [84.7–99.5]	97.0% [95.5–98.1]	97.0% [95.5–98.1]	0.724
* vanA/vanB *	61	39	37	680	24	2	94.9% [83.1–98.6]	96.6% [95.0–97.7]	96.5% [94.9–97.6]	0.720
* dfrA1/dfrA5/sul1/sul2 *	85	72	70	656	15	2	97.2% [90.4–99.2]	97.8% [96.3–98.7]	97.7% [96.3–98.6]	0.875
* ermB/ermC/mefA *	74	59	57	667	17	2	96.6% [88.3–99.1]	97.5% [96.0–98.5]	97.4% [96.0–98.4]	0.840
* mecA *	15	12	12	728	3	0	100.0% [75.8–100.0]	99.6% [98.8–99.9]	99.6% [98.8–99.9]	0.886
* blaIMP/blaNDM/blaVIM group *	25	12	12	718	13	0	100.0% [75.8–100.0]	98.2% [97.0–98.9]	98.2% [97.0–98.9]	0.640
* qnrA/qnrS (Combined) *	66	46	44	675	22	2	95.7% [85.5–98.8]	96.8% [95.3–97.9]	96.8% [95.3–97.8]	0.769

AST+, phenotypically non-susceptible (intermediate or resistant [I/R]) according to the applicable parent-study organism–antimicrobial mapping; AST−, phenotypically susceptible (S); TP, true positive; TN, true negative; FP, molecular-positive/AST-negative; FN, molecular-negative/AST-positive; PPA, positive percent agreement; NPA, negative percent agreement; OPA, overall percent agreement; CI, confidence interval; κ, Cohen’s kappa; AMR, antimicrobial resistance. Molecular AMR target–phenotypic AST mappings and interpretation rules are provided in Supplementary Table S1. Molecular reporting groups were not necessarily mutually exclusive. The estimates represent genotype–phenotype agreement with the parent-study phenotypic AST classifications and not independent molecular confirmation of individual resistance genes. Molecular detection of a resistance determinant does not itself establish phenotypic non-susceptibility, and absence of a targeted determinant does not establish phenotypic susceptibility. *OXA-51* was retained within the assay AMR reporting architecture as an A. baumannii-associated oxacillinase target. Because *blaOXA-51-like* genes are intrinsic to A. baumannii, detection of this target alone should not be interpreted as evidence of carbapenem resistance or as an independent predictor of the carbapenem phenotype.

## Data Availability

Due to the sensitive nature of the clinical data and patient confidentiality requirements, the datasets used and/or analyzed during the current study are not publicly available. However, they are available from the corresponding author on reasonable request, provided that the request complies with relevant ethical guidelines and data protection regulations.

## Supplementary Materials

The following supporting information can be downloaded at: https://www.mdpi.com/article/10.3390/cimb48090930/s1.
