# Supplementary material for: Species-Level Reconfiguration and Retrospective Bridging Assessment of a Multiplex PCR Panel for Complicated and Recurrent Urinary Tract Infections
Source: Curr Issues Mol Biol. 2026 Sep 11;48(9):930. doi: 10.3390/cimb48090930 (PMC13605023; doi:10.3390/cimb48090930)
Supplement: Supplementary file 1 [file cimb-48-00930-s001.zip › cimb-4527885-supplementary.pdf]

Supplementary Table S1. Molecular AMR Target–Phenotypic AST Mapping Applied in the Genotype–Phenotype Agreement Analyses

Molecular AMR target / reporting group	Assay version(s) included in analysis	Resistance mechanism represented	Organism(s) applicable to phenotypic comparison	Prespecified phenotypic AST comparator	Molecular-positive / AST-positive classification rule	Interpretation / important limitation
CTX-M Group 1	V2 and V5	Extended-spectrum β-lactamase (ESBL)	Primarily Enterobacterales, including *E. coli*, *Klebsiella* spp., *Proteus* spp., and other applicable Enterobacterales	Parent-study expanded-spectrum cephalosporin/ESBL-compatible phenotype used for the prespecified genotype–phenotype comparison	Molecular positive when CTX-M Group 1 was detected; AST positive when the corresponding isolate met the parent-study prespecified β-lactam resistance classification	CTX-M is a major ESBL determinant. Phenotypic resistance may occur through other ESBL/AmpC mechanisms; therefore, phenotype positivity is not equivalent to direct CTX-M confirmation.
blaKPC	V2 and V5	Class A serine carbapenemase	Primarily Enterobacterales; other Gram-negative isolates only where the prespecified organism–phenotype rule applied	Parent-study carbapenem non-susceptibility phenotype mapped to the KPC reporting target	Molecular positive when *blaKPC* was detected; AST positive according to the prespecified carbapenem-resistance classification for the applicable isolate	KPC detection strongly supports a carbapenemase mechanism, but carbapenem resistance may occur through mechanisms other than KPC.
blaNDM	V2 and V5	Class B metallo-β-lactamase	Enterobacterales and other applicable Gram-negative organisms	Parent-study carbapenem non-susceptibility phenotype mapped to *blaNDM*	Molecular positive when *blaNDM* was detected; AST positive according to the applicable prespecified carbapenem-resistance classification	NDM commonly produces broad β-lactam resistance. Phenotypic susceptibility cannot be inferred solely from absence of *blaNDM*.
OXA-48	V2 and V5	Class D carbapenemase	Primarily Enterobacterales	Parent-study carbapenem/carbapenemase-compatible phenotype mapped to OXA-48	Molecular positive when OXA-48 was detected; AST positive according to the prespecified carbapenem phenotype	OXA-48-like enzymes may produce variable carbapenem MICs, including relatively modest elevations for some agents; therefore, genotype–phenotype discordance is biologically plausible and the AST phenotype is not a direct confirmation of OXA-48.

OXA-51	V2 and V5; interpreted separately as an exploratory association	Intrinsic *A. baumannii* OXA-51-like determinant	*Acinetobacter baumannii* only	Exploratory comparison with the parent-study *A. baumannii* carbapenem phenotype; no one-to-one phenotypic AST equivalent exists for OXA-51 detection	Molecular positive when the OXA-51-like target was detected; phenotypic classification based on the applicable parent-study carbapenem result for *A. baumannii*. This comparison was interpreted descriptively rather than as validation of a direct resistance predictor.	Critical caveat: *bla*OXA-51-like genes are intrinsic to *A. baumannii* and may support species identification. Detection alone does not establish carbapenem resistance; consequently, this analysis should not be interpreted as validation of OXA-51 as a stand-alone predictor of carbapenem resistance.
tetB/tetM	V2 and V5	Tetracycline efflux (*tetB*) and ribosomal protection (*tetM*)	Only organisms for which an applicable tetracycline-class AST result was available	Parent-study tetracycline-class phenotype prespecified for comparison with the *tetB/tetM* reporting group	Molecular positive when either reportable determinant within the predefined *tetB/tetM* group was detected; AST positive according to the applicable tetracycline-class resistance classification	Molecular detection indicates a recognized tetracycline-resistance mechanism; clinical interpretation remains organism dependent. The phenotypic comparator is not specific for either *tetB* or *tetM*.
ACT/MIR/FOX/ACC group	V2 and V5	AmpC β-lactamase-associated mechanisms	Applicable Enterobacterales	Parent-study β-lactam phenotype prespecified for comparison with the ACT/MIR/FOX/ACC AmpC-associated reporting group	Molecular positive when a reportable determinant within the predefined ACT/MIR/FOX/ACC group was detected; AST positive according to the corresponding prespecified β-lactam phenotype	Phenotypic β-lactam resistance is not specific for ACT/MIR/FOX/ACC determinants and may also reflect chromosomal AmpC expression, ESBL production, permeability changes, or combinations of mechanisms. Phenotype positivity therefore does not constitute confirmation of an AmpC-family gene.
PER-1/VEB-1/GES-1 group	V2 and V5	Selected class A β-lactamase/ESBL-	Applicable Gram-negative isolates	Parent-study expanded-spectrum cephalosporin/ESBL-compatible phenotype	Molecular positive when PER-1, VEB-1, or GES-1 was detected within the	The mapped cephalosporin phenotype is not specific for PER-1, VEB-1, or GES-1 and may result from other β-

		associated determinants		prespecified for this reporting group	predefined reporting group; AST positive according to the mapped ESBL-associated phenotype	lactamase mechanisms. Grouped molecular detection also does not identify which determinant is responsible for the phenotype.
vanA/vanB	V2 and V5	Acquired glycopeptide resistance	*Enterococcus* spp.	Vancomycin non-susceptibility	Molecular positive when *vanA* or *vanB* was detected within the reporting group; AST positive when the corresponding enterococcal isolate was classified as vancomycin non-susceptible in the parent-study AST result	*vanA/vanB* are major acquired VRE determinants, but genotype and phenotype should still be reported as distinct measurements.
dfrA1/dfrA5/sul1/sul2	V2 and V5	Trimethoprim and sulfonamide resistance	Applicable bacterial uropathogens for which TMP-SMX AST was available	Trimethoprim–sulfamethoxazole non-susceptibility	Molecular positive when a reportable determinant within the predefined *dfr/sul* reporting group was detected; AST positive when the applicable isolate was classified as TMP-SMX non-susceptible	Because several alternative mechanisms can affect TMP-SMX susceptibility, gene absence does not establish phenotypic susceptibility.
ermB/ermC/mefA	V2 and V5	Macrolide-resistance determinants; *erm* genes may mediate MLS-type resistance, whereas *mefA* primarily mediates macrolide efflux	Applicable Gram-positive organisms with an interpretable macrolide phenotype	Parent-study macrolide-resistance phenotype prespecified for this molecular reporting group	Molecular positive when *ermB*, *ermC*, or *mefA* was detected within the predefined group; AST positive according to the corresponding parent-study macrolide classification	Clinical relevance depends on organism and drug. The grouped target contains mechanisms with different resistance spectra and should not be interpreted as a general UTI-treatment susceptibility marker or as direct prediction of lincosamide susceptibility.
mecA	V2 and V5	Altered penicillin-binding protein–mediated	*Staphylococcus aureus* and applicable	Parent-study methicillin-resistance phenotype for the relevant *Staphylococcus* isolate	Molecular positive when *mecA* was detected; AST	*mecA* is a canonical determinant of methicillin resistance in *Staphylococcus*

		methicillin resistance	coagulase-negative staphylococci		positive when the applicable staphylococcal isolate met the parent-study methicillin-resistance classification	spp. Interpretation is most direct when the relevant staphylococcal organism can be linked to the *mecA* result. In polymicrobial specimens without explicit organism–gene linkage, specimen-level *mecA* detection should not automatically be attributed to a specific species.
blaIMP/blaNDM/blaVIM group	V2 and V5	Metallo-β-lactamase group	Applicable Enterobacterales and non-fermenting Gram-negative isolates	Parent-study carbapenem/MBL-compatible phenotype prespecified for the grouped reporting target	Molecular positive when any reportable IMP, NDM, or VIM determinant within the grouped channel was detected; AST positive according to the applicable prespecified carbapenem phenotype	Molecular positivity was defined at the grouped reporting level. This group is not mutually exclusive with the standalone *blaNDM* target; the same specimen may contribute to both reporting categories. Results from the grouped MBL channel and standalone *blaNDM* analysis should therefore not be summed across categories.
qnrB	V2 only; omitted from V5	Plasmid-mediated quinolone resistance	Primarily applicable Enterobacterales	Parent-study fluoroquinolone phenotype prespecified for the V2 *qnrB* comparison	Molecular positive when *qnrB* was detected; AST positive according to the mapped fluoroquinolone resistance classification	qnr determinants contribute to reduced quinolone/fluoroquinolone susceptibility but may not independently produce phenotypic non-susceptibility at clinical breakpoints. Phenotypic resistance may also arise through chromosomal target mutations, efflux, and other mechanisms. The analysis therefore represents genotype–phenotype association rather than direct prediction of fluoroquinolone susceptibility. Omission from V5 was an architectural decision and does not imply biological irrelevance.

qnrS	V2 as an individual target; incorporated into the combined V5 *qnrA/qnrS* channel	Plasmid-mediated quinolone resistance	Primarily applicable Enterobacterales	Parent-study fluoroquinolone phenotype prespecified for the relevant qnr comparison	In V2, molecular positive when *qnrS* was detected; AST positive according to the mapped fluoroquinolone phenotype. V5 results were evaluated through the consolidated *qnrA/qnrS* reporting channel.	qnr-mediated reductions in susceptibility may remain below clinical resistance breakpoints, and fluoroquinolone resistance can occur through mechanisms unrelated to qnr. Accordingly, genotype and phenotype are not expected to correspond one-to-one.
qnrA1/qnrA2	V2 as individual qnrA reporting targets; incorporated into the combined V5 *qnrA/qnrS* channel	Plasmid-mediated quinolone resistance	Primarily applicable Enterobacterales	Parent-study fluoroquinolone phenotype prespecified for the relevant qnrA comparison	In V2, molecular positive when a reportable *qnrA1/qnrA2* signal was detected; AST positive according to the mapped fluoroquinolone phenotype. V5 results were evaluated through the consolidated *qnrA/qnrS* channel.	qnrA detection may contribute to reduced fluoroquinolone susceptibility without independently producing clinical resistance, while alternative chromosomal and efflux mechanisms can produce phenotypic resistance in the absence of qnrA.
Combined *qnrA/qnrS*	V5 consolidated reporting target	Plasmid-mediated quinolone resistance	Primarily applicable Enterobacterales	Same parent-study fluoroquinolone phenotype used for the applicable qnr genotype–phenotype comparison	Molecular positive if the combined V5 channel detected a reportable *qnrA* or *qnrS* signal; phenotypic positive according to the mapped fluoroquinolone AST classification	

AST, antimicrobial susceptibility testing; AST+, phenotypically non-susceptible; AST−, phenotypically susceptible; AMR, antimicrobial resistance; MIC, minimum inhibitory concentration; ESBL, extended-spectrum β-lactamase; MBL, metallo-β-lactamase; TMP-SMX, trimethoprim–sulfamethoxazole. For all genotype–phenotype comparisons, the original parent-study categorical AST interpretations were retained: intermediate or resistant results (I/R) were classified as AST-positive/non-susceptible, whereas susceptible results (S) were classified as AST-negative. MIC values were not retrospectively reinterpreted using a new breakpoint standard. Molecular and phenotypic results represent distinct biological measurements; detection of a resistance determinant does not necessarily establish phenotypic non-susceptibility, absence of a targeted determinant does not establish phenotypic susceptibility, and organism–gene linkage was not assumed in polymicrobial specimens unless explicitly established.

Table S2: Pathogen Composition Mapping (V2 vs. V5) with Scientific Evidence Support

V2 Pathogen Target	V5 Pathogen Target(s)	Status / Action	Structural and Clinical Rationale	Scientific Evidence Supporting Inclusion / Exclusion and Clinical Relevance	Supporting References
Escherichia coli	*Escherichia coli*	Unchanged	Retained as a principal urinary pathogen and to preserve continuity with the clinically evaluated V2 configuration.	Evidence-supported major uropathogen across community and healthcare-associated UTI populations.	11–14
Pseudomonas aeruginosa	*Pseudomonas aeruginosa*	Unchanged	Retained because of its relevance to healthcare-associated, catheter-associated, and structurally complicated urinary infections.	Associated with healthcare exposure, catheterization, biofilm formation, and antimicrobial resistance.	15–17
Acinetobacter baumannii	*Acinetobacter baumannii*	Unchanged	Retained as a clinically important healthcare-associated and multidrug-resistant organism.	Lower prevalence in routine UTI but important because of antimicrobial resistance and limited treatment options.	15, 18
Staphylococcus aureus	*Staphylococcus aureus*	Unchanged	Retained because urinary detection may have clinical significance in selected high-risk and healthcare-associated populations.	Associated with catheterization, hospitalization, and possible bloodstream infection.	18–20
Staphylococcus saprophyticus	*Staphylococcus saprophyticus*	Unchanged	Retained to preserve broad uropathogen coverage.	Established urinary pathogen, although particularly associated with uncomplicated UTI.	12, 13
Citrobacter koseri	*Citrobacter koseri*	Unchanged	Retained for coverage of opportunistic Enterobacterales in high-risk populations.	Documented healthcare-associated urinary pathogen.	21
Serratia marcescens	*Serratia marcescens*	Unchanged	Retained because of its association with instrumentation and healthcare-associated urinary infection.	Biofilm formation and healthcare-associated infection relevance.	15, 21
Streptococcus pyogenes	*Streptococcus pyogenes*	Unchanged	Retained for continuity with the V2 configuration and broad organism coverage, despite its low expected urinary prevalence.	Urinary infection is uncommon; therefore, its retention does not imply high expected clinical yield.	21
Streptococcus agalactiae	*Streptococcus agalactiae*	Unchanged	Retained because of relevance in selected vulnerable populations.	Recognized urinary isolate with particular relevance in pregnancy and older adults.	11, 21
Mycoplasma genitalium	*Mycoplasma genitalium*	Unchanged	Retained as a selected urogenital target for context-dependent interpretation rather than as a conventional cUTI pathogen.	*M. genitalium* is an established cause of urethritis and may be relevant in selected symptomatic patients; its detection should be interpreted within the clinical and sexual-health context.	38
Ureaplasma urealyticum	*Ureaplasma urealyticum*	Unchanged	Retained as a selected urogenital target for patients in whom clinical context supports additional molecular evaluation.	Colonization is common and routine detection or treatment is not universally recommended; therefore, molecular detection requires clinical correlation and should not independently establish urinary infection.	39

Citrobacter freundii/braakii	*Citrobacter freundii/braakii*	Notation Update	Nomenclature/reporting refinement without a change in the underlying target group.	Relevant Enterobacterales with resistance considerations.	15, 22
Enterococcus faecium/faecalis	*E. faecium* / *E. faecalis*	Updated (Unbundled)	Separated to provide species-level reporting for organisms with different epidemiological and resistance profiles.	*E. faecium* is more frequently associated with multidrug and vancomycin resistance than *E. faecalis*.	23–25
Klebsiella pneumoniae/oxytoca	*K. pneumoniae* / *K. oxytoca*	Updated (Unbundled)	Separated to improve species-level reporting and avoid ambiguity inherent to composite detection.	Species differ in epidemiology and resistance mechanisms.	15, 22, 26
Proteus mirabilis/vulgaris	*P. mirabilis* / *P. vulgaris*	Updated (Unbundled)	Separated to improve organism-level reporting.	*P. mirabilis* is strongly associated with urinary infection and stones; species-specific resistance characteristics support differentiation.	27, 28
Staphylococcus (CoNS)	*S. epidermidis* / *S. haemolyticus* / *S. lugdunensis*	Updated (Expanded)	Expanded to provide species-level resolution among CoNS with differing clinical significance.	*S. lugdunensis* has greater virulence than many other CoNS.	19, 20
Candida spp.	*C. albicans* / *C. glabrata* / *C. parapsilosis* / *C. tropicalis*	Updated (Expanded)	Expanded to provide species-level identification rather than a generic *Candida* result.	Species differ in epidemiology and antifungal susceptibility; clinical interpretation requires correlation with symptoms and specimen context.	29, 30
Gardnerella vaginalis	None	Omitted	Omitted to prioritize targets more directly aligned with the intended cUTI/rUTI application and available multiplex capacity.	*Gardnerella* is an important component of the genitourinary microbiota and has been reported in selected urinary infections; omission therefore reflects intended-use prioritization rather than absence of potential urinary relevance.	40
Neisseria gonorrhoeae	None	Omitted	Removed to distinguish the cUTI/rUTI-focused panel from comprehensive STI testing.	Clinically important STI pathogen but outside the principal intended cUTI/rUTI target set.	12, 2
Chlamydia trachomatis	None	Omitted	Removed to focus the panel on the principal urinary pathogens and AMR determinants relevant to the intended application.	Clinically important STI pathogen; dedicated STI testing remains appropriate when indicated.	12, 2
Trichomonas vaginalis	None	Omitted	Removed to maintain focus on the intended cUTI/rUTI application.	Primarily associated with genital infection rather than complicated bacterial UTI.	2
None	*Providencia stuartii*	New Addition	Added because of its relevance to catheter-associated and healthcare-associated urinary infection.	Particularly associated with long-term catheterization and antimicrobial resistance.	31
None	*Morganella morganii*	New Addition	Added to broaden coverage of resistant Enterobacterales relevant to complicated urinary infection.	Associated with healthcare-associated and catheter-associated infections and intrinsic resistance mechanisms.	15, 22

None	*Corynebacterium urealyticum*	New Addition	Added because of its association with selected complicated urinary syndromes and fastidious growth.	Associated with alkaline-encrusted cystitis and may be difficult to recover using routine culture.	32
None	*Combined Aerococcus urinae/A. sanguinicola target*	New Addition	Added to increase coverage of selected fastidious Gram-positive urinary organisms.	Associated with urinary infection and invasive disease, particularly in older adults.	2
None	*Enterobacter cloacae*	New Addition	Added for coverage of clinically relevant AmpC-associated Enterobacterales.	Important healthcare-associated organism with inducible AmpC resistance.	15, 22
None	*Klebsiella aerogenes*	New Addition	Added for coverage of an Enterobacterales species with clinically relevant resistance mechanisms.	Formerly *Enterobacter aerogenes*; associated with healthcare-associated infection and AmpC-mediated resistance.	22
None	*Bacillus atrophaeus*	New Addition: Internal Process Control	Added as a non-clinical molecular process control to monitor assay-processing performance; not included as a pathogen-expansion target.	Used to monitor molecular assay/process performance.	33

Table S3: AMR Target Mapping from V2 to V5, Modification Category, and Supporting Scientific Rationale

V2 AMR Target / Group	V5 AMR Target	Status / Action	Technical and Stewardship Rationale	Scientific Evidence Supporting Inclusion / Exclusion and Clinical Relevance	Supporting References
CTX-M Group 1	*bla*KPC	Unchanged	Retained because of its clinical relevance as a major carbapenemase determinant.	KPC is an important carbapenemase mechanism among CRE and has implications for antimicrobial selection and infection-control management.	18, 26
blaKPC	CTX-M Group 1	Unchanged	Retained as a clinically relevant ESBL-associated target.	CTX-M enzymes are major ESBL determinants and are frequently identified among resistant Enterobacterales.	26, 34
blaNDM	*bla*NDM	Unchanged	Retained as a clinically important carbapenemase determinant.	NDM-producing organisms are associated with broad β-lactam resistance and limited therapeutic options.	18, 26
OXA-48	OXA-48	Unchanged	Retained because of its relevance to carbapenemase-mediated resistance.	OXA-48-like carbapenemases are clinically important carbapenem-resistance mechanisms.	18, 26
OXA-51	OXA-51	Unchanged	Retained as an existing *A. baumannii*-associated oxacillinase target within the assay AMR reporting architecture.	*blaOXA-51-like genes are intrinsic to A. baumannii; detection alone does not establish carbapenem resistance and should not be interpreted as an independent predictor of the carbapenem phenotype.*	18, 26
tetB/tetM	*tetB* / *tetM*	Unchanged	Retained with standardized target representation.	Established tetracycline-resistance determinants; clinical significance depends on the organism and treatment context.	18
ACT/MIR/FOX/ACC	ACT / MIR / FOX / ACC	Unchanged	Retained to preserve detection of selected AmpC-associated resistance determinants.	AmpC-associated mechanisms are clinically relevant because they may reduce susceptibility to extended-spectrum cephalosporins.	22, 26
PER-1/VEB-1/GES-1	PER-1 / VEB-1 / GES-1	Unchanged	Retained for broader ESBL/resistance surveillance.	These resistance determinants are less prevalent in some US settings than CTX-M but remain clinically recognized mechanisms.	26
vanA/vanB	*vanA* / *vanB*	Unchanged	Retained for detection of acquired vancomycin resistance determinants in Enterococcus.	*vanA* and *vanB* are major determinants of acquired glycopeptide resistance.	23–25

dfrA1/dfrA5/sul1/sul2	*dfrA1* / *dfrA5* / *sul1* / *sul2*	Unchanged	Retained with standardized gene-level nomenclature.	These determinants contribute to trimethoprim-sulfamethoxazole resistance; clinical interpretation remains organism- and phenotype-dependent.	35
ermB/ermC/mefA	*ermB* / *ermC* / *mefA*	Unchanged	Retained for molecular surveillance of selected macrolide-, lincosamide-, and streptogramin-resistance mechanisms.	Established resistance mechanisms among Gram-positive organisms; clinical relevance depends on the organism detected.	18
mecA	*mecA*	Unchanged	Standardized molecular nomenclature and reporting.	*mecA* is a major molecular determinant of methicillin resistance in *Staphylococcus* species.	18–20
blaIMP/blaNDM/blaVIM group	*bla*IMP / *bla*NDM / *bla*VIM	Unchanged	Standardized *bla* nomenclature without an intended change in target class.	IMP, NDM, and VIM are clinically important metallo-β-lactamase determinants.	18, 26
qnrS	*qnrS* / *qnrA*	Consolidated	Combined with *qnrA* into the V5 *qnrA/qnrS* reporting channel to retain representation of both target families within a single reporting channel under the fixed multiplex/reporting capacity. This architectural consolidation does not establish analytical equivalence or superiority relative to the separate V2 targets..	*qnrA* and *qnrS* are recognized plasmid-mediated quinolone-resistance determinants. The consolidation represents an architectural change and does not by itself establish equivalent or superior V5 performance.	18
qnrA1, *qnrA2*	*qnrS* / *qnrA*	Consolidated	Incorporated with *qnrS* into the combined V5 *qnrA/qnrS* reporting channel as part of the revised multiplex architecture.	*qnrA* and *qnrS* contribute to plasmid-mediated quinolone resistance. Independent validation is required to establish the analytical performance of the combined V5 target.	18
qnrB	None	Omitted	Not carried forward within the fixed multiplex/reporting capacity after prioritization of the combined *qnrA/qnrS* channel. The decision was not based on inadequate V2 performance or evidence of lesser biological relevance of *qnrB*.	*qnrB remains a recognized plasmid-mediated quinolone-resistance determinant; its omission reflects V5 panel architecture and target prioritization and should not be interpreted as biological or clinical irrelevance.*	18
